# Complete genome of a cereulide-producing *Bacillus mobilis* strain

**DOI:** 10.1128/mra.00782-25

**Published:** 2025-11-24

**Authors:** Hendrik Frentzel, Ylanna Kelner-Burgos, Pascal Witt, Maria Borowiak, Carlus Deneke

**Affiliations:** 1 Department Biological Safety, German Federal Institute for Risk Assessment (BfR) 27652, Berlin, Germany; University of Maryland School of Medicine, Baltimore, Maryland, USA

**Keywords:** *Bacillus cereus*, toxin, emetic, plasmid

## Abstract

Hitherto, reported cereulide-producing strains of the *Bacillus cereus* group belonged to the *panC* clades 3 or 6 and shared the highest average nucleotide identity (ANI) with *B. paranthracis* or *B. mycoides*. Here, we report an emetic strain assigned to the *panC* clade 2 and the ANI species *B. mobilis*.

## ANNOUNCEMENT

The *Bacillus cereus* group contains strains that are able to produce the emetic toxin cereulide, which causes food poisoning. The genetic prerequisite for cereulide production is the *cesHPTABCD* gene cluster. Most described emetic *B. cereus* group strains are mesophilic, belong to the phylogenetic *panC* clade 3, share the highest average nucleotide identity (ANI) with the species *B. paranthracis,* and carry the *ces* genes on a 270 kb plasmid (1–3). Very rarely, psychrotolerant emetic strains are detected (*panC* clade 6 and highest ANI with *B. mycoides*) (4–6).

The emetic strain BfR-BA-01700 described in this study was isolated in accordance with ISO 7932-2004 by spread plating on mannitol egg yolk polymyxin agar, followed by incubation for 24 h to 48 h at 30°C, from a sample of meat salad in Germany in 2021 by a local food control authority. The strain belongs to the *panC* clade 2 and shares the highest ANI with *B. mobilis*. The ability of the strain to produce cereulide has been described previously (7). The *cesHPTABCD* gene cluster is located on a 349,073 bp plasmid. Using NCBI BlastN, the sequence of the classical 270 kb pCER270 plasmid (strain AH187; GenBank accession: DQ889676.1) showed a query coverage of 90% and an identity of 99.96% to the 349,073 bp plasmid.

The genomic analyses comprised short-read Illumina and long-read Oxford Nanopore Technologies sequencing. For DNA extraction, the strain was streaked from a −80°C glycerol culture on sheep blood agar. Material from a single colony was used to inoculate 3 mL of brain heart infusion broth (Oxoid), which was incubated at 30°C and 150 rpm overnight. From 1 mL of this culture, DNA was extracted with the DNeasy Blood & Tissue Kit (Qiagen, Hilden, Germany) and used for sequencing without further processing or size selection of the DNA.

For short-read sequencing, the Nextera DNA Flex kit (Illumina, San Diego, CA, USA) was applied, and sequencing was performed in 2×149 bp cycles on an Illumina NextSeq 500, resulting in 2,832,646 raw reads. The pipeline AQUAMIS v1.2.0-18-g1317d15 was used for trimming, quality check, and *de novo* assembly of sequencing reads (8).

For ONT long-read sequencing, the SQK-RBK110.96 kit (Oxford Nanopore Technologies, Oxford, UK) was used to prepare a sequencing library. The library was sequenced with a FLO-MIN106 flow cell and a MinIon Mk1C device. The generated data were subsequently basecalled using guppy v6.0.1 in SUP mode, resulting in 141,748 raw reads (https://nanoporetech.com/community). The obtained data were quality controlled and assembled using the MilongA pipeline v.1.0.1 (https://gitlab.com/bfr_bioinformatics/milonga). There, reads were trimmed with Porechop v0.2.4 (https://github.com/rrwick/Porechop) and further processed and evaluated using NanoFilt v2.8.0 and NanoStat v1.5.0 (9). The short-read and the long-read data sets were *de novo* assembled and circularized using Unicycler v0.4.8, including Pilon for polishing (10–12).

Based on this assembly, we performed further characterizations with the pipeline BakCharak v3.1.6 (13). The annotation was completed with PGAP (14–16) at the NCBI.

Characterization results, along with details about read quality control, assembly, and applied tools, are summarized in Table 1.

**TABLE 1:** Genetic characteristics of the emetic *B. mobilis* strain and applied tools and databases

	Strain BfR-BA-01700	Tools and databases (all tools were used with default settings)
short-read quality control	2,755,852 reads (after trimming); 88% ≥ Q30	AQUAMIS v1.2.0-18-g1317d15 (8) utilizing fastp v0.19.5 (17)
long-read quality control	71,429 reads (after trimming and filtering); N50: 6,313 bp	Porechop v0.2.4 (https://github.com/rrwick/Porechop), NanoFilt v2.8.0 & NanoStat v1.5.0 (9)
assembly	GCA_033841795.2 (complete, seven contigs) CP185965.1: chromosome; 5,277,139 bp; circular; %GC: 35.5 CP185966.1: plasmid; 349,073 bp; circular; %GC: 34 CP185967.1: plasmid; 66,276 bp; circular; %GC: 32 CP185968.1: plasmid; 52,852 bp; circular; %GC: 36.5 CP185969.1: plasmid; 41,501 bp; circular; %GC: 32.5 CP185970.1: plasmid; 32,086 bp; circular; %GC: 35.5 CP185971.1: plasmid; 3,166 bp; circular; %GC: 33	MiLongA pipeline 1.0.1 (https://gitlab.com/bfr_bioinformatics/milonga), Unicycler v0.4.8 (11, 12), Pilon v1.24 (10), platon v1.6 (18), Geneious Prime 2020.2.2
species based on ANI	ANI coverage: 92% ANI value: 99.9 best ANI reference strain: *B. mobilis* 0711P9-1 (type strain)	BakCharak pipeline v3.1.6 (13) utilizing FastANI v.1.33 (19) and the typestrain database from BTyper v.3.4.0 (20)
*panC* clade	clade 2 (legacy as well as adjusted clade)	BakCharak pipeline v3.1.6 (13) utilizing abricate v1.0.1 (https://github.com/tseemann/abricate) and the *panC* databases of (20)
signature of 16s rRNA gene and *cspA* gene	16s rRNA gene: mesophilic signature; *cspA* gene: mesophilic signature	BakCharak pipeline v3.1.6 (13) utilizing bakta v1.9.3 (21) and the mesophilic and psychrotolerant signature sequences according to (22) for *cspA* and (23) for the 16 s rRNA gene
MLST	784	BakCharak pipeline v3.1.6 (13) utilizing MLST v2.23.0 (https://github.com/tseemann/mlst) and the PubMLST Database: 2024-01-16 (24)
*cesHPTABCD* gene cluster	location: 349,073 bp plasmid coverage (%) and identity (%) with respective gene sequences from reference strain AH187: *cesH*: 100 and 100 *cesP*: 100 and 100 *cesT*: 100 and 100 *cesA*: 100 and 100 *cesB*: 100 and 100 *cesC*: 100 and 99.89 *cesD*: 100 and 100	BakCharak pipeline v3.1.6 (13) utilizing abricate v1.0.1 (https://github.com/tseemann/abricate) and the VFDB (SetB): 2022-08-26 (25)

## Data Availability

All data are available under BioProject PRJNA1037718 and BioSample SAMN38195819. Sequencing raw reads were deposited in the NCBI Sequence Read Archive (SRA) under the accession numbers SRX28127605 (ONT data) and SRX28127604 (Illumina data). The complete genome sequence is available at NCBI GenBank under accession number GCA_033841795.2.
